# Relationship Between Hybrid Arts-Based CBT-CP Intervention and Personality Dimensions in Patients with Non-Malignant Chronic Pain: Evidence from a Non-Randomized Control Trial

**DOI:** 10.3390/healthcare13121440

**Published:** 2025-06-16

**Authors:** Asimina Kalmanti, Vasiliki Yotsidi, Athanasios Douzenis, Vasiliki Moraiti, Aikaterini Malafanti, Ioannis Michopoulos

**Affiliations:** 1 2nd Department of Psychiatry, Medical School, National and Kapodistrian University of Athens, “Attikon” University Hospital, 1 Rimini Str, 12462 Athens, Greece; thandouz@med.uoa.gr (A.D.); imihopou@med.uoa.gr (I.M.); 2 Department of Psychology, Panteion University of Social and Political Sciences, 136 Syggrou Ave, 17671 Athens, Greece; v.yiotsidi@panteion.gr (V.Y.); katerina.malafanti@gmail.com (A.M.); 3 Department of Occupational Therapy, University of West Attica, Ag. Spyridonos Str., Egaleo, 12243 Athens, Greece; valia.moraiti@gmail.com

**Keywords:** non-malignant chronic pain, personality, arts-based CBT-CP group intervention, mindfulness, non-randomized control trial

## Abstract

**Background**: Optimal coping with chronic pain (CP) has a positive impact on minimizing the barriers to patients’ quality of life. Mindfulness-based approaches have been shown to improve emotional regulation and coping strategies in CP management, promoting a greater acceptance of pain and reducing psychological distress. Given that personality traits may influence the adjustment to chronic pain, this study aimed to investigate whether specific personality dimensions, based on Cloninger’s model of temperament–character dimensions, affect the enrolment and the response to pain treatment in an innovative hybrid arts-based CBT-CP group intervention for patients with non-malignant CP. **Methods**: A pre-and-post assessment design was implemented in a non-randomized control trial. A total number of 100 outpatients at a University Pain Management Unit were allocated through self-selection in either an arts-based CBT-CP group intervention (*N* = 50) or a treatment-as-usual (TAU) control group (*N* = 50). All participants completed the Brief Pain Inventory (BPI), the Orbach and Mikulincer Mental Pain Scale (OMMP), the Tolerance for Mental Pain Scale (TMPS), and the Temperament and Character Inventory (TCI-140). The assessment took place at baseline and at the end of the intervention, after a 10-week period. The statistical analyses included a *t*-test for independent samples, Chi-square, and linear regression analyses. **Results**: At baseline, the arts-based CBT-CP intervention group had a higher score in the novelty seeking character dimension (*M* = 64.04; *SD* = 9.56), whereas the TAU group was found to have higher scores in self-directedness (*M* = 74.34; *SD* = 11.22) and self-transcendence (*M* = 51.42; *SD* = 6.61). The arts-based CBT-CP group reported a lower loss of control (*M* = 22.94; *SD* = 6.70) and higher belief in the ability to cope with pain (*M* = 21.10; *SD* = 3.76) after the intervention, compared to the control group. Self-transcendence was found to be a significant predictor of average pain as well as of patients’ belief in their ability to cope with pain. **Conclusions**: The current study provides practice-based evidence suggesting that an arts-based CBT-CP group intervention is a promising treatment for non-malignant CP. Personality dimensions affect patients’ enrolment and response to pain treatment. Furthermore, integrating mindfulness-based strategies within such interventions may further enhance treatment outcomes by fostering acceptance, improving coping mechanisms, and reducing the emotional burden associated with chronic pain.

## 1. Introduction

Non-malignant chronic pain (CP) is a common, complex, and distressing health problem with significant adverse personal and social implications [1,2]. A holistic CP management that is theoretically driven by the biopsychosocial pain model and encompasses a variety of therapeutic strategies has been repeatedly shown to be both clinically useful and cost-effective [3,4,5,6]. Such a broad treatment approach seeks to provide evidence-based therapies according to personalized treatment plans through the combination of various pharmaceutical and psychosocial treatment options, and the continuation of systematic and comprehensive care delivery [7,8]. Along these lines, it is important to shed light on the role of patients’ personality traits to their response to CP treatment in order to provide tailor-made care services, especially when new interventions are incorporated into empirically supported treatments.

Cognitive Behavioral Therapy for Chronic Pain (CBT-CP) resonates with the current perspectives of CP management, because it conceptualizes chronic pain as resulting from complex interactions between several biological, emotional, social, and behavioral processes, and aims to reduce the traumatic pain experience by modifying the respective psychosocial factors [9]. Although CBT-CP has become the first-line psychological intervention for CP, as an empirically validated treatment according to the National Institute of Health and Care Excellence Guidelines [10] and the Society of Clinical Psychology (Division 12 of the American Psychological Association; see https://div12.org/diagnosis/chronic-or-persistent-pain/ accessed on 28 March 2025), there is still room for further improvement due to cases of lack of treatment response to CBT and/or relapse from treatment [11]. In alignment with the growing interest in integrative therapies, mindfulness-based interventions (MBIs) have also been shown to enhance the biopsychosocial model by fostering present-moment awareness and improving patients’ emotional regulation and coping mechanisms [12]. In addition, accumulated evidence supports the added value of using creative and arts-based therapeutic techniques in psychological treatments for CP management [13,14,15,16]. Indeed, a review of studies on the application of art therapy to chronic pain management showed that art interventions have a clinically significant effect on reducing pain intensity, improving mental well-being, and increasing optimism, resilience, and psychosocial adjustment [17]. More recently, a CBT-CP intervention that incorporated components of Creative Arts Therapies (CATs) into CBT-CP was found to contribute to traditional pain management as a complementary CP treatment method [18]. The implementation of conceptual principles of CATs and the promotion of psychological change through arts-based techniques (i.e., visualization, writing and drawing exercises, and creative expression of emotions), combined with CBT-CP strategies (i.e., psychoeducation, problem-solving, positive affirmation, cognitive restructuring, relaxation training, mindfulness exercises, and relapse prevention), appear to be promising since they provide a holistic and integrative framework for addressing cognitive, affective, and behavioral aspects of CP more effectively [18,19,20].

Although personality traits have been found to play an important role in the onset of and adjustment to CP [21,22], there is still limited data available regarding how specific temperament and character dimensions may affect patients’ response to non-malignant CP treatment, particularly in cases of multi-component approaches that address patients’ perceptions, emotions, and behavioral responses to pain. Interestingly enough, the relationship between personality and pain has been explored throughout the ages [23,24]. Greek philosophers, such as Plato and Aristotle, believed that pain arose not only from physical stimuli but also from emotions in the soul [25], thus highlighting the ancient holistic mind–body tradition that is reflected in the modern biopsychosocial paradigm. Along these lines, the relationship between affective disturbance and physical pain may be framed in terms of predisposing, precipitative, perpetuating, and exacerbating factors of CP, as well as consequences from CP [26,27].

Given the impact of personality on better coping and dealing with CP in creative ways, further research into the interplay between personality traits, interventions including arts, cognitive behavioral approaches and mindfulness, and CP may offer deeper insights. Studies have shown that mindfulness can mitigate the influence of negative affectivity, increase psychological flexibility, and improve adaptive coping in CP patients [28,29]. Exploring how these benefits interact with individual differences, such as temperament and character dimensions, could make a significant contribution to personalized treatment approaches for CP patients [30,31,32,33,34].

In line with the biopsychosocial approach to CP, Cloninger’s (1987) seven-dimensional model of personality conceptualizes personality as including four temperament and three character dimensions, with the former reflecting genetically determined personality characteristics, and the latter reflecting traits affected by learning processes in life. Particularly, temperament traits include harm avoidance, novelty seeking, reward dependence, and persistence. These temperament dimensions manifest as automatic emotional responses to life experiences and reflect neurotransmitter systems in the brain. Character traits include self-directedness, cooperativeness, and self-transcendence. Cloninger has suggested that character dimensions are affected by the environment and life experiences, and thus character matures until late adulthood [35,36,37].

Psychological well-being depends on the development of three personality dimensions, that is (a) autonomy and life purpose as stemmed from self-directedness, (b) positive relations with others derived from cooperativeness, and (c) personal growth and self-actualization emerged from self-transcendence [38]. High self-transcendence scores have been associated with positive emotions and well-being [36,39,40]. Self-transcendence experience is considered multifaceted in itself, composed of mindfulness, flow, and self-transcendent emotions, such as awe, peak moments, and mystical experiences. The antecedents and attributes of self-transcendence are organized into five interrelated domains: creativity, relationships, introspection, contemplation, and spirituality [41,42,43,44]. Spirituality is a determinant of psychological well-being, independently of health-related behavior, and there is increasing evidence to support the inclusion of spiritual factors as an important component in the assessment and treatment of CP [45,46]. Of note, spiritual intervention programs have received relatively little attention within the field of pain medicine itself [47,48,49].

Hence, the role of Cloninger’s personality dimensions in understanding the underlying principles and mechanisms of individuals’ well-being [37,50] provides a promising theoretical background to incorporate when addressing CP management. Temperament and character traits have been associated with resilience and lifelong well-being, as well as with how people respond to their environment, in general [32]. Given the diverse nature of CP and the need for comprehensive long-term CP management, the application of Cloninger’s holistic personality theory to examine how personality traits may affect patients in a new therapeutic intervention as well as their response to treatment will allow for customized assessment and treatment processes to be developed.

To the best of our knowledge, this is the first study to examine the role of patients’ personality profiling in an integrated arts-based CBT-CP group intervention for people suffering from non-malignant chronic pain. Specifically, this study aimed to investigate whether specific personality (temperament and character) dimensions according to Cloninger’s model affect the enrolment and the response to treatment. Such knowledge would inform our efforts to better respond to CP by means of an individualized and patient-tailored therapeutic approach that would provide diverse treatment options. Based on the objectives of the study, the respective research questions are delineated as follows:

1. Which patients’ personality (i.e., temperament and character) dimensions are associated with their enrolment in an arts-based CBT-CP intervention compared to treatment-as-usual?
2. How are patients’ personality (i.e., temperament and character) dimensions associated with physical pain, mental pain, and tolerance for mental pain?
3. Which patients’ personality (i.e., temperament and character) dimensions predict better response to treatment, based on pre–post measurements of physical pain, mental pain, and tolerance for mental pain among those receiving an arts-based CBT-CP intervention compared to treatment-as-usual?

## 2. Method

### 2.1. Participants

The sample consisted of 100 outpatients with non-malignant CP who were enrolled in the Pain Management Unit of a University General Hospital in Greece. The inclusion criteria of participants were the following: (a) to fall within the 18–60 years age range; (b) to be diagnosed with non-malignant CP of any etiology; and (c) to have agreed not to receive any other psychosocial treatment during the course of the study. The exclusion criteria applied to those who (a) were not fluent in the Greek language; and/or (b) were diagnosed with any mental health disorder, based on a psychiatric interview prior to the participation in the study.

### 2.2. Measures

According to the Initiative on Methods, Measurement, and Pain Assessment in Clinical Trials (IMMPACT) [51], the following scales were used in the study.

### 2.3. The Brief Pain Inventory (BPI)

The BPI is a 9-item self-completed scale that evaluates patients’ experience of physical pain in a number of different pain states. It is a reliable tool for monitoring both the effect of pain and the treatment of pain in terms of a patient’s functional ability or disability over time [52]. Cronbach’s alphas range from 0.77 to 0.91. The BPI has been validated in Greek (G-BPI) with adequate internal consistency (α = 0.80) [53].

### 2.4. The Orbach and Mikulincer Mental Pain Scale (OMMP)

The OMMP consists of 44 self-rated items that conceptualize mental pain as a perception of negative emotions. The items of the OMMP are divided into nine factors, namely irreversibility, loss of control, narcissistic wounds, emotional flooding, freezing, self-estrangement, confusion, social distancing, and emptiness [54]. The OMMP has been translated and validated in Greek. For the Greek version, Cronbach’s alphas have been found to be 0.96 for the total scale and ranging from 0.74 to 0.91 for the subscales, with social distancing having poor reliability (α = 0.39), as it was also the case in the original version [55].

### 2.5. The Tolerance for Mental Pain Scale (TMPS) 

The 20-item TMPS was developed to assess the tolerance of psychological pain. The TMPS measures three facets of tolerance for psychological pain that reflect respective theoretical perspectives in the literature: surfeit of the pain, belief in the ability to cope with the pain, and containing the pain. The TMPS has been translated and validated in Greek. The Greek version showed adequate internal consistency estimates. Cronbach’s alphas were 0.84 for the total scale, and 0.87, 0.76, and 0.72 for the three subscales, respectively [56].

### 2.6. The Temperament and Character Inventory (TCI-140)

The TCI-140 is a well-known personality inventory based on Cloninger’s model consisting of seven subscales that measure four temperament and three character dimensions, as separate personality variants. Temperament subscales include harm avoidance, novelty seeking, reward dependence, and persistence. Character subscales include self-directedness, cooperativeness, and self-transcendence [39,57]. The TCI-140 has been translated and validated in Greek, showing adequate psychometric properties and retaining the initial factorial structure. The Cronbach’s alpha scores of the Greek version ranged from 0.51 to 0.83 [58].

### 2.7. Procedure

The research protocol of this study was approved (RN#EBD2106/6-2-2017) by the Research Ethics Board of the Medical School of the National and Kapodistrian University of Athens. This study was a non-randomized control trial and was conducted from February 2017 to November 2019. Pre–post measurements took place at baseline (T0) and at the end of the arts-based CBT-CP intervention (T1), after a 10-week period. All outpatients with non-malignant CP complaints, who were new admissions at the Pain Management Unit of a University General Hospital, were referred by their physician to the research group to assess their eligibility for participation in this study. From a total number of 160 new admissions, 115 patients were accepted to participate in this study, after a psychiatric assessment interview took place so as to exclude individuals with comorbid conditions. Fifteen of them withdrew after the baseline assessment and prior to the intervention, without attending any sessions. From the resulting sample of 100 patients, 50 patients accepted to receive the arts-based CBT-CP intervention and formed the intervention group, whereas the remaining 50 patients declined and preferred to follow the treatment-as-usual (TAU) approach, which included routine check-ups and medication management with a primary care physician, thus forming the control group.

A pre–post assessment design was implemented in this study. The goal of treatment for the arts-based CBT-CP intervention was stated to all patients as follows: “to learn active use of coping strategies to deal with pain, as well as skills that would help you in your daily life and in adopting healthy lifestyles” [18]. All participants signed an informed consent form prior to their participation in this study and completed the questionnaires in a single session while they were in the waiting room at the Pain Management Unit. In the case that a patient experienced a headache or had any other difficulty, a new appointment was arranged to ensure the accuracy and quality of responses. The arts-based CBT-CP groups were run by an experienced mental health occupational therapist who was trained in CBT within a certified training program and had 10-year working experience with CP patients. Every experimental group consisted of 8–10 participants (except an initial pilot one with 4 patients), who attended 10 weekly structured and manualized two-hour sessions, in total. No attrition occurred. More details about the structure and content of the arts-based CBT-CP intervention were provided in a previous paper [18].

Particularly, the arts-based CBT-CP intervention was jointly based on the extensive modification of Thorn’s CP treatment manual [59] and the trauma-related framework of expressive art therapies [60], including drawing and expressive writing as a means of arts engagement. Each session incorporated CBT-CP principles (e.g., cognitive defusion), mindfulness-based techniques, and visual and narrative activities to promote attention-focusing skills, creative expression, and positive adjustment to chronic pain. The intervention encompassed an “initial treatment phase” (2 sessions) introducing the treatment goals and tasks and building a rapport among the participants and the therapist. The next six sessions (sessions 3–8) were the “main treatment phase of CBT skills building”, when the participants worked through their thoughts, emotions, and current coping strategies to deal with chronic pain by drawing (e.g., drawing the lived experience of the pain) and expressive writing activities (e.g., self-compassion letter). The last so-called “discharge phase” of the intervention (sessions 9–10) aimed at establishing expressive communication as a constructive coping strategy and consolidating the gains of the whole treatment process. A detailed description of the various modules, the creative arts activities, and the theoretical foundations of the intervention is portrayed in the link of the supplementary material of a previous paper [18] (https://www.psychiatriki-journal.gr/documents/psychiatry/Kalmanti%202021_Supplementary%20material.pdf, accessed on 28 March 2025).

### 2.8. Statistical Analysis

Quantitative variables were presented as mean values, standard deviations, and minimum/maximum values across the total population and between groups. Categorical variables were expressed as absolute and relative frequencies (*N*, %). Patient characteristics at baseline were compared using the non-parametric Mann–Whitney U test and Chi-square or Fisher’s exact test, when necessary. Pearson *r* and Spearman *rho* correlation coefficients were also used. In order to compare the temperament and character inventory scores on the seven personality dimensions between patients in the intervention group and those in the control group, independent t-tests were used for normally distributed personality dimensions, whereas Mann–Whitney *U* tests were applied for non-normally distributed dimensions. When significant differences were found on a personality dimension, additional analyses on the subscales of the particular dimension were performed. Linear regression analyses were conducted to examine the associations between the temperament and character inventory dimensions and pre–post intervention differences for physical pain, mental pain, and tolerance for mental pain scores. All models were adjusted for age and gender. The statistical tests were two-sided and were performed at a 0.05 significance level. Post-hoc power calculations were conducted using the G*Power program (version 3.1.9.4) [61] based on the sample size achieved (*N* = 100; 50 per group). For independent samples *t*-tests, the power to detect medium effect sizes (Cohen’s *d* = 0.50) was approximately 90%, while the power for small effect sizes (*d* = 0.20) was around 32%. For regression analyses with seven predictors, power calculations for detecting medium effect sizes (*f*^2^ = 0.15) exceeded 99%, and power for small effect sizes (*f²* = 0.02) was approximately 41%. These values indicate that the study was well-powered to detect medium to large effects, while small effects may have gone undetected. Specifically, variables with ** power > 0.80 ** (e.g., *Novelty Seeking*, *Self-Transcendence*, and *Loss of Control Post*) were well-powered. Statistical analyses were performed using IBM SPSS Statistics 25.0 [62].

## 3. Results

### 3.1. Sociodemographic and Non-Malignant Chronic Pain Characteristics

Patients’ demographic characteristics, such as gender, age, education, and marital status, as well as the types of pain they experienced according to the ICD-11 non-malignant chronic pain classification are presented in Table 1. The vast majority of participants were females (84%) in both groups, with a tendency of younger participants to be included in the intervention group (48.9 ± 9.29 vs. 55.8 ± 3.67, *p* < 0.001), indicating that younger patients were more willing to test a novel therapeutic approach. No other significant differences were found between the two groups. Similarly, there were no significant differences on non-malignant chronic pain classification [63].

### 3.2. Comparisons Between the Arts-Based CBT-CP Group and the TAU Group as a Function of Personality Dimensions and Non-Malignant Chronic Pain Variables

As shown in Table 2, concerning the personality dimensions, there were statistically significant differences at the baseline measurement between the intervention group and the control group for novelty seeking, self-directedness, and self-transcendence. Specifically, participants in the control group were found to have higher scores in self-directedness and self-transcendence, whereas participants of the intervention group had a higher score in novelty seeking. As mentioned above, according to Cloninger’s model, novelty seeking comprises a genetically determined temperament dimension, which manifests as an automatic emotional response to life challenges. Thus, the patients with increased levels of novelty seeking could be deemed to have a propensity for accepting the challenge of participating in a new CP treatment approach. On the other hand, the increased self-directedness and self-transcendence scores of patients in the control group may reflect their attempt to preserve autonomy and well-being by being reluctant to enroll in an unfamiliar treatment experience, which potentially could jeopardize their emotional and spiritual status quo.

At the same time, as indicated in Table 3, prior to the intervention, the intervention group had a significantly higher total pain-related interference score. Perhaps this may have also played a role in patients who chose the arts-based CBT-CP intervention rather than the TAU intervention, as they may have not seen any positive effect from previous chronic pain management interventions. Yet, although after the intervention the mean pain-related interference score was slightly lower in the intervention group compared with the control group, the difference between the two groups was not statistically significant.

Furthermore, the average pain prior to and after the intervention was found to be significantly higher in the intervention group compared with the control group (Table 3). On the other hand, the intervention group had a significantly lower loss of control score after the intervention compared with the control group. Also, with regard to the participants’ belief in their ability to cope with the pain, although patients in the intervention group believed that they were less able to cope with pain prior to the intervention, they reported having a higher belief in their ability to cope with pain after the intervention compared with the control group. Thus, the arts-based CBT-CP intervention led to positive changes in certain personality traits associated with coping with pain (i.e., lower levels of loss of control along with a higher belief in the ability to cope with the pain), although not directly reducing the intensity of pain itself.

### 3.3. Associations Between Personality Dimensions and Non-Malignant Chronic Pain Variables

Harm avoidance and self-transcendence had a moderate positive correlation with loss of control in the intervention group before the intervention. The correlation between harm avoidance and loss of control was significantly higher in the intervention group before the intervention (see Table 4). That is, for patients who enrolled in the intervention group, the higher the harm avoidance and self-transcendence personality dimensions the more they experienced loss of control of pain before the intervention. Again, these results may indicate the patients’ tendency of openness to a new experience due to a reduced extent of defense against chronic pain (i.e., loss of control) and a rather positive attitude towards learning from their own painful experience (i.e., self-transcendence) rather than simply avoiding its harmful impact (i.e., harm avoidance).

As indicated in Table 5, no significant associations were observed between personality dimensions and loss of control in both groups after the intervention. However, the correlation between self-directedness and loss of control was significantly higher (moderate) in the intervention group compared to the control group after the intervention. This finding shows that acquiring a sense of autonomy and life purpose as stemmed from self-directedness does not exclude losing control of pain. Rather, it seems that the arts-based CBT-CP intervention assisted participants in accepting the loss of control of pain as a contributing factor to self-directedness.

### 3.4. Prediction of Non-Malignant Chronic Pain Variables by Personality Dimensions

Linear regression analyses were conducted for the prediction of differences in physical pain, mental pain, and tolerance for mental pain by personality (temperament and character) variables. Self-transcendence was found to be a significant predictor of average pain and of the belief in the ability to cope with the pain for the total sample of patients (see Table 6). That is, a rather spiritual personality dimension was found to predict low levels of pain and the belief in the ability to cope with pain, a finding that may open new avenues in interdisciplinary healthcare research.

## 4. Discussion

The aim of this study was to examine whether specific personality (temperament and character) dimensions according to Cloninger’s model affect the enrolment and the response to an arts-based CBT-CP intervention of patients with non-malignant chronic pain. The results showed that, before the intervention, the patients in the intervention group had a higher score in the novelty seeking personality dimension, whereas their levels of loss of control were associated with harm avoidance compared with the TAU group. Additionally, before the intervention, the average pain levels in the arts-based CBT-CP group were significantly higher, and patients believed that they were less able to cope with pain compared with the control group. Similarly, after the intervention, the average pain levels were found to be significantly higher in the arts-based CBT-CP group. However, after the intervention the arts-based CBT-CP group had a significantly lower loss of control score and reported a higher belief in their ability to cope with pain as compared with the control group. This finding aligns with evidence suggesting that therapeutic approaches fostering self-awareness and acceptance, such as mindfulness-based interventions (MBIs), can enhance self-regulation and reduce perceived helplessness in the context of CP [11,12]. Notably, the character dimension of self-transcendence was a significant predictor of average pain as well as the patients’ belief in their ability to cope with chronic pain for both groups. These findings are consistent with the literature suggesting that higher levels of self-transcendence and mindfulness are linked to greater psychological flexibility and improved emotional resilience in individuals with chronic pain [39,57].

Additionally, our findings are in line with recent studies using Cloninger’s model of personality which suggest that higher harm avoidance and lower self-directedness appear to be the most distinguishing personality features of CP patients. High harm avoidance refers to patients’ tendency to be pessimistic, sensitive to criticism and punishment, and to seek reassurance. Low self-directedness often manifests as low motivation, difficulty in setting meaningful goals, and problems in adaptive coping. Such a personality profile is associated with a fear-avoidance response to pain, which is likely to contribute to the development and persistence of CP. Consequently, patients with higher harm avoidance and lower self-directedness may be more vulnerable, presenting a low threshold at which pain is perceived as threatening, and they may also find it difficult to stop chronic rumination, take action to overcome avoidance, and engage in more active coping behaviors [61,64,65]. Cloninger argues that high self-directedness and low harm avoidance are powerful predictors of well-being and life satisfaction [43,53]. Indeed, the findings of our study showed that arts-based therapeutic models for CP, such as the arts-based CBT-CP intervention, may help CP patients to overcome fear and avoidance, and instead feel more empowered to deal with their chronic health problems, thus increasing their resilience [66].

Along with the personality dimensions that could be an integral part of any CP management, this study sheds light on how treatment could be customized to adequately “match” patients’ clinical and demographic variables with specific therapeutic techniques (i.e., arts-based CBT techniques vs. medicine-based interventions). Patients who participated in the hybrid arts-based CBT-CP group were more likely to be younger and novelty seekers compared with the group of patients who preferred to receive a medical treatment for CP. The temperament dimension of novelty seeking (i.e., a tendency toward exhilaration or excitement in response to cues of potential reward or relief of punishment) may explain the willingness of the patients in the arts-based CBT-CP group to participate in the intervention and might compensate for other personality factors indicative of vulnerability. Cloninger’s model suggests that dimensions of temperament are heritable and that novelty seeking and harm avoidance are closely related to the behavioral approach system and behavioral inhibition system, respectively. In addition, the model suggests a link between certain temperamental dimensions and specific neurotransmitters, that is, between novelty seeking and dopamine, and between harm avoidance and serotonin [67,68]. Despite their higher average pain and total pain-related interference, patients in the arts-based CBT-CP group expressed a preference for adaptive and creative coping strategies. Interestingly, although their levels of pain did not differ significantly at the end of the intervention, those patients reported less loss of control and increased belief in their ability to cope with pain, having also enhanced their tolerance for mental pain. These findings run parallel to those of other studies which suggest that acceptance and cognitive distraction have a positive impact on pain tolerance [68,69]. Thus, despite the lack of decrease in physical pain, meaning-making of and coping with pain appear as important resilience factors against suffering, which is possible to achieve depending on the improvement of a person’s positive attitude, management resources, as well as choices and commitments related to resilience [19,65,66,69,70].

An intriguing finding of this study was that the character dimension of self-transcendence was a significant predictor of low levels of pain and the belief in the ability to cope with pain. Self-transcendence refers to transpersonal character strengths and to the interest in finding something superior to individual existence [71]. According to Cloninger’s model, self-transcendence manifests as a superior personality trait, like compassion, morality, and a thorough understanding of art and culture. Self-transcendence can be regarded not only as a personality trait, but also as a psychological state, a developmental process, a value orientation, a motivational force, and finally a worldview that produces a spiral effect in which meaning, virtue, and happiness interact and are cumulatively built [71,72]. Given that self-transcendence indicates individual differences in self-concepts, attitudes, and goals that influence voluntary choices, intentions, and the meaning and salience of what is experienced in life, research has shown a positive association of self-transcendence with an optimistic adaptive coping style [73,74]. Our findings imply that addressing self-transcendence through spirituality and creativity as central elements of this fundamental character strength can enhance patients’ adjustment to a major stressor, such as CP. This adjustment is facilitated by replacing a ‘threat’ perspective of pain—which triggers harm avoidance and loss of control—with a ‘challenge’ perspective that promotes resilient coping, quality of life, and well-being. Studies indicate that practices enhancing self-transcendence, including mindfulness-based interventions, can lead to reduced emotional reactivity and greater acceptance of pain, fostering psychological resilience [75]. Given the key role of self-transcendence in managing CP effectively, addressing its negative effects through arts, creativity, cognitive processing, and group interaction may stimulate cognitive openness and psychological flexibility. This aligns with research suggesting that mindfulness and other attention-based practices can refocus the cognitive system on positive and previously overlooked aspects of experience, reducing the intensity of negative appraisals and encouraging a sense of control and agency [76]. Re-aligning attention in this manner creates the possibility of positively re-appraising the CP condition, potentially resulting in higher levels of experienced meaningfulness. These outcomes reflect the integration of emotional awareness and cognitive reappraisal strategies, as described in frameworks emphasizing mindfulness and positive psychology [77].

Several mechanisms of change embedded with arts-based interventions explain how the hybrid arts-based CBT-CP intervention might have led to better coping with chronic pain. Psychological mechanisms, such as distraction, emotional catharsis, embodiment, and symbolic processing, which lie at the core of art therapy, may have facilitated non-verbal emotional processing and acceptance of previously disavowable thoughts and emotions [78]. Through creative expression, patients are helped to recognize their potential and increase insight by means of a combined action towards perception, sensation, experiential body experience, creativity, and meaning [79]. Creative arts-based activities (e.g., visualization, writing and drawing exercises, and creative expression of emotions) contribute to distraction from pain and enhancement of personal pain management resources, such as visualization and relaxation. Also, the patients can release intense emotions related to pain, their personal history, or their relationships with others. Instead of merely talking about their emotional experience, they express internal trauma-related conflicts or painful emotions through metaphors and symbolism. By this way, the patients’ need for emotional expression is channeled through the creation of embodied images, which articulate emotions in a visual way. These embodied images contain personal feelings, thoughts and ideas and “hold” them until the patients manage to work through and integrate them in a meaningful and constructive way [80]. Hence, by encouraging imagination, expression, awareness, and an enhanced relationship with the world through embodied practices [81], art therapy expands the boundaries between therapy, creativity, and experience. The findings of our study resonate with the respective findings of previous empirical and phenomenological studies on how creativity assists in reframing pain and fostering agency [13,14,15,16,17].

Given that the arts and mindfulness-based interventions could provide a framework for understanding recovery, a major strength of the present study was that it contributes to filling the gap of exploring the link between personality and creativity in real clinical conditions, by showing that clinical characteristics and patients’ personality are relevant to the patient-related treatment outcomes [2,82,83,84]. Moreover, this study offers new practice-based evidence suggesting that self-transcendence is a broad and complex clinical construct that may enrich the current therapeutic objectives of CBT-CP, such as fear-avoidance and self-efficacy. Another strength of this study was that all the intervention groups were moderated by the same mental health professional with expertise at implementing both CBT and arts interventions, minimizing thus the therapist’s confounding effects, while there was systematic supervision by a psychiatrist specialized in CBT throughout the implementation of this study. Other strengths include the participants’ retention and adherence to the treatment program, the comparable groups at baseline, and that the outcomes of this study cover a range of different types of non-malignant CP.

On the other hand, the limitations of this study need to be considered. First, this was a non-randomized clinical trial, because the patients themselves chose the group to which they would like to participate. Although this was the most salient limitation of this study, a non-randomized design was considered necessary as one of our aims was to investigate enrolment in an arts-based CBT-CP intervention as a function of personality variables. Also, the sample was primarily White female patients with non-malignant CP, a fact that limits the generalizability of the findings. Further research based on a randomized controlled trial design in different cultural contexts with diverse ethnic, age, and gender populations is necessary to expand the clinical validity of these results. In addition, no follow-up procedures were undertaken to explore the potential long-term effects of the arts-based CBT-CP intervention. This is of particular importance considering the unchanged pain levels despite other improvements, as a main finding of this study, which may be due to the specific duration of the intervention (i.e., 10 sessions) instead of the common 12–20 sessions of CBT treatment. Although the arts-based CBT-CP intervention was adjusted to take into account the increased patient attrition in CP management, a long-term intervention might have been necessary for a chronic health condition, such as chronic pain, to be improved.

Notwithstanding these limitations, the present study lays bare the importance of integrating creative arts therapies in CBT and mindfulness-based approaches in CP treatment as a means to increase patients’ ability to cope with chronic pain and ameliorate the psychological burden that stems from CP. Such an integrative intervention can also help health professionals from different disciplines (e.g., medical doctors, psychiatrists, psychologists, occupational therapists, etc.) to better cooperate with each other while working with patients with chronic pain. Considering that treating chronic pain can be quite demanding and expensive especially for minorities [85], the combination of creative arts therapies with empirically supported treatments (e.g., CBT-CP) along with the provision of medical services could enhance the quality of CP management and minimize the cost of treatment. Providing tailor-made CP treatment based on patients’ personality profiling could also contribute to less costly and more effective treatment alternatives. Particularly, the finding of the present study about the role of self-transcendence in predicting the levels of pain as well as the patients’ belief in their ability to cope with chronic pain brings to the fore the importance of interdisciplinary research and clinical practice and the potential role of spirituality in healthcare. Usually, health professionals are not trained in spirituality or other aspects of self-transcendence, thus lacking the opportunity to incorporate them in their clinical work. Adding the role of personality (i.e., temperament and character) dimensions in the healthcare professionals’ training would promote holistic CP management driven by the biopsychosocial pain model and improve the assessment and treatment of patients with non-malignant chronic pain.

Nevertheless, the question of whether personality traits may predispose an individual to CP or whether the personality characteristics emerge after the onset of CP remains an issue to be examined in future research. Investigating the relationship between personality dimensions and several clinical variables (i.e., age of onset of CP, duration of CP, previous CP treatment, patient’s motivation, patient’s satisfaction, etc.) as well as specific components of the therapeutic intervention (i.e., group vs. individual delivery of treatment, specific techniques, etc.) is an additional array of further research. For example, arts-based techniques in this study included visualization, writing and drawing exercises, and creative expression of emotions. However, we did not examine which one of them may have had a differential effect, or whether alternative artistic activities (e.g., visual, performative, or narrative) could have been of added clinical value for patients with chronic pain. Finally, future studies that would examine the efficacy of the arts-based CBT-CP intervention with the participation of more mental health clinicians (i.e., CBT-trained psychologists) and in different contexts are necessary for building robust evidence about the outcomes of such an integrative intervention in CP management.

## 5. Conclusions

The present study contributed to the existing literature by providing evidence of the clinical utility of personality (temperament and character) dimensions for a customized treatment planning and delivery for patients with non-malignant CP. Harm avoidance, self-directedness, and self-transcendence appear to play an important role in CP treatment. The findings of this study indicate that assessing and fostering self-transcendence through integrative therapeutic interventions that recognize the added health value of creativity and combine arts with CBT-CP and mindfulness-based exercises may help patients to better cope with chronic pain and disability. Future clinical research and practice may expand to include such integrative and holistic therapeutic interventions that promote health and well-being, by considering patients’ personality dimensions in the assessment and implementation of CP management interventions.

## Figures and Tables

**Table 1:** Sociodemographic characteristics and non-malignant chronic pain classification across groups.

Variables	Intervention Group (*N* = 50)	Control Group (*N* = 50)	*t*-Statistic (*p*-Value)	Effect Size (η)^2^
Gender (female)	42 (84%)	42 (84%)	1	
Age	48.9 ± 9.29 (22–60)	55.8 ± 3.67 (40–60)	−3.898 (<0.001) ^b^	0.15
Education (years)	12.1 ± 4.35 (6–18)	11.8 ± 3.53 (6–18)	−0.140 (0.889) ^b^	0.00
Marital status			3.337 (0.068) ^a^	
*Married*	29 (64.4%)	39 (81.3%)		
*Living alone*	16 (35.6%)	9 (18.7%)		
ICD-11 non-malignant chronic pain classification [45]			5.709 (0.320) ^a^	
*Headache/orofacial pain*	5 (10%)	2 (4%)		
*Musculoskeletal pain*	16 (32%)	19 (38%)		
*Neuropathic pain*	9 (18%)	11 (22%)		
*Chronic posttraumatic/postsurgical pain*	0 (0%)	3 (6%)		
*Visceral pain*	1 (2%)	0 (0%)		
*Mixed pain*	19 (38%)	15 (30%)		

*Note.* Μ ± SD (range) or N (%) are displayed, as appropriate. ^a^ χ^2^ or Fisher’s exact test. ^b^ Mann–Whitney *U* test.

**Table 2:** Differences in the temperament and character inventory dimensions between groups.

Dimensions	*M* (*SD*)		*t*-Statistic (*p*-Value)	Effect Size (Cohen’s *d*)
	Intervention Group	Control Group		
Harm Avoidance	61.96 (12.46)	59.46 (13.78)	−0.951 (0.344)	−0.19
Novelty Seeking	64.04 (9.56)	41.78 (5.29)	−14.402 (<0.001) ^a^	−2.88
Reward Dependence	66.22 (12.19)	68.44 (9.61)	1.011 (0.314)	0.20
Persistence	70.94 (11.54)	68.96 (9.83)	−0.924 (0.358)	−0.18
Self-Directedness	68.14 (14.94)	74.34 (11.22)	2.346 (0.021) ^a^	0.47
Cooperativeness	77.02 (10.82)	76.82 (9.99)	−0.096 (0.924)	−0.02
Self-Transcendence	45.84 (8.62)	51.42 (6.61)	3.631 (<0.001) ^a^	0.73

*Note.* ^a^ *p* < 0.05.

**Table 3:** Differences in the Brief Pain Inventory (BPI), the Orbach and Mikulincer Mental Pain Scale (OMMP), and the Tolerance for Mental Pain Scale (TMPS) dimensions between groups before and after the intervention.

Variables	*M* (*SD*)		*z*-Statistic (*p*-Value)	Effect Size (η^2^)
	Intervention Group	Control Group		
Total pain-related interference with functioning—pre (BPI)	6.45 (2.45)	5.20 (1.50)	−4.074 (<0.001) ^a^	0.17
Total pain-related interference with functioning—post (BPI)	4.72 (2.47)	5.55 (1.32)	−1.616 (0.106)	0.03
Average pain—pre (BPI)	6.26 (1.89)	3.90 (1.49)	−5.901 (<0.001) ^a^	0.35
Average pain—post (BPI)	4.74 (2.27)	3.66 (1.47)	−2.861 (0.004) ^a^	0.08
Loss of control—pre (OMMP)	28.72 (6.92))	31.38 (6.61	−1.678 (0.093)	0.03
Loss of control—post (OMMP)	22.94 (6.70)	37.94 (4.41)	−7.926 (<0.001) ^a^	0.63
Belief in the ability to cope with the pain—pre (TMPS)	19.10 (4.06)	20.54 (8.14)	−2.657 (0.008) ^a^	0.07
Belief in the ability to cope with the pain—post (TMPS)	21.10 (3.76)	13.7 (5.31)	−6.435 (<0.001) ^a^	0.42

*Note.* ^a^ *p* < 0.05. Mann–Whitney U tests.

**Table 4:** Spearman *rho* correlations between the temperament and character inventory dimensions and the loss of control score (OMMP) across groups before the intervention and differences of correlation coefficients between the groups.

Variables	Spearman *rho* (*p*-Value)		Fisher’s *z* (*p*-Value)
	Intervention Group	Control Group	
Harm Avoidance	0.341 (0.015) *	−0.057 (0.693)	−1.999 (0.046) *
Novelty Seeking	0.071 (0.624)	0.099 (0.495)	0.137 (0.891)
Reward Dependence	−0.177 (0.220)	0.120 (0.406)	1.452 (0.147)
Persistence	−0.215 (0.133)	0.007 (0.964)	1.093 (0.275)
Self-Directedness	−0.275 (0.053)	−0.161 (0.264)	0.581 (0.561)
Cooperativeness	−0.066 (0.651)	−0.079 (0.586)	−0.063 (0.949)
Self-Transcendence	0.307 (0.030) *	0.240 (0.093)	−0.351 (0.725)

*Note.* * *p* < 0.05 level.

**Table 5:** Spearman rho correlations between the temperament and character inventory dimensions and the loss of control score (OMMP) across groups after the intervention and differences of correlation coefficients between the groups.

Variables	Spearman *rho* (*p*-Value)		Fisher’s *z* (*p*-Value)
	Intervention Group	Control Group	
Harm Avoidance	0.351 (0.012) *	0.027 (0.854)	−1.646 (0.100)
Novelty Seeking	0.180 (0.212)	0.14 (0.331)	−0.199 (0.842)
Reward Dependence	−0.081 (0.576)	0.079 (0.584)	0.777 (0.437)
Persistence	−0.235 (0.100)	−0.046 (0.75)	0.938 (0.348)
Self-Directedness	0.380 (0.006) *	−0.252 (0.077)	−3.188 (0.001) *
Cooperativeness	−0.167 (0.248)	−0.023 (0.874)	0.706 (0.480)
Self-Transcendence	−0.09 (0.535)	0.224 (0.117)	1.542 (0.123)

*Note.* * *p* < 0.05 level.

**Table 6:** Prediction of difference in physical pain, mental pain and tolerance for mental pain by TCI by the temperament and character inventory dimensions in the total sample.

Variables	Unstandardized Coefficients		Standardized Coefficients	*t*-Statistic	*p*-Value
	B	Std. Error	Beta		
DV ^a^: Diff ^b^ (Total Pain-Related Interference with Functioning Score—BPI)					
Novelty Seeking	−0.014	0.012	−0.141	−1.198	0.234
Harm Avoidance	0.004	0.008	0.035	0.445	0.657
Reward Dependence	−0.003	0.010	−0.024	−0.313	0.755
Persistence	−0.010	0.009	−0.082	−1.195	0.235
Self-Directedness	0.002	0.008	0.015	0.187	0.852
Cooperativeness	0.001	0.010	0.007	0.093	0.926
Self-Transcendence	−0.014	0.013	−0.084	−1.122	0.265
Group	−1.754	0.332	−0.641	−5.279	<0.001
DV ^a^: Diff ^b^ (Average Pain Score—BPI)					
Novelty Seeking	−0.015	0.017	−0.148	−0.921	0.359
Harm Avoidance	−0.007	0.011	−0.062	−0.581	0.563
Reward Dependence	0.006	0.013	0.045	0.422	0.674
Persistence	0.004	0.012	0.032	0.343	0.733
Self-Directedness	−0.013	0.011	−0.128	−1.155	0.251
Cooperativeness	0.003	0.014	0.024	0.220	0.827
Self-Transcendence	−0.048	0.017	−0.282	−2.756	0.007
Group	−1.014	0.461	−0.365	−2.200	0.030
DV ^a^: Diff ^b^ (Loss of Control Score—OMMP)					
Novelty Seeking	0.065	0.082	0.104	0.792	0.430
Harm Avoidance	0.004	0.056	0.006	0.069	0.945
Reward Dependence	−0.024	0.067	−0.031	−0.357	0.722
Persistence	0.029	0.061	0.037	0.487	0.627
Self-Directedness	−0.005	0.057	−0.008	−0.090	0.928
Cooperativeness	−0.002	0.072	−0.003	−0.034	0.973
Self-Transcendence	0.046	0.087	0.044	0.534	0.595
Group	−13.329	2.292	−0.786	−5.817	<0.001
DV ^a^: Diff ^b^ (Belief in the Ability to Cope with the Pain Score—TMPS)					
Novelty Seeking	−0.062	0.072	−0.123	−0.869	0.387
Harm Avoidance	−0.040	0.049	−0.078	−0.832	0.408
Reward Dependence	0.064	0.058	0.102	1.096	0.276
Persistence	0.006	0.053	0.009	0.113	0.911
Self-Directedness	−0.071	0.049	−0.139	−1.429	0.157
Cooperativeness	−0.004	0.062	−0.006	−0.065	0.948
Self-Transcendence	−0.193	0.076	−0.230	−2.549	0.013
Group	8.721	1.994	0.641	4.374	<0.001

*Note.* ^a^: DV = dependent variable, ^b^: diff = (post—pre). BPI = Brief Pain Inventory. OMMP = Orbach and Mikulincer Mental Pain Scale. TMPS = Tolerance for Mental Pain Scale.

## Data Availability

The original contributions presented in this study are included in the article. Further inquiries can be directed to the corresponding author.
